# Glomerular expression of matrix metalloproteinases in systemic lupus erythematosus in association with activity index and renal function

**DOI:** 10.1080/0886022X.2019.1591998

**Published:** 2019-04-11

**Authors:** Konstantinos N. Adamidis, Maria-Emmanouela Kopaka, Constantina Petraki, Evangelia Charitaki, Theophanis Apostolou, Christallenia Christodoulidou, Nikoleta Nikolopoulou, Alexandra Giatromanolaki, Vassilios Vargemesis, Ploumis Passadakis

**Affiliations:** a Private Hemodialysis Unit, “Bionephros”, Athens, Greece;; b Department of Pathology, Metropolitan Hospital, Athens, Greece;; c Department of Nephrology, General Hospital of Chania, Chania, Greece;; d Department of Nephrology, Evangelismos General Hospital, Athens, Greece;; e Department of Pathology, University Hospital of Alexandroupoli, Alexandroupolis, Greece;; f Department of Nephrology, University Hospital of Alexandroupoli, Alexandroupolis, Greece

**Keywords:** Lupus nephritis, matrix metalloproteinases, MMP

## Abstract

**Purpose:** The aim of this study was to examine the expression of matrix metalloproteinases (MMPs) MMP-1, MMP-2, MMP-3, MMP-9, and their specific tissue inhibitor TIMP-1 in kidney biopsies of patients with lupus nephritis (LN) and to investigate the relationship between MMPs, activity index, and renal function at the time of kidney biopsy.

**Methods:** We performed immunohistochemistry with monoclonal antibodies against MMP-1, MMP-2, MMP-3, MMP-9, and TIMP-1 in 58 kidney-biopsy specimens with LN (according to the 2004 ISN/RPS classification) and eight specimens from normal kidney tissue. We used clinical data of 36 patients at the time of kidney biopsy to evaluate the association between MMPs expression and renal function.

**Results:** We found increased MMP-1, MMP-2, and MMP-3 expression in LN glomeruli and a significant correlation with the activity features, with higher activity index score and worse renal function (*p* < .001). In particular, we have noticed a significant correlation of MMP-1 with leukocyte influx (OR:16.5 95%CI 4.3–62.5 *p* < .001), and MMP-3 with glomerular hypercellularity (OR:18.6 95%CI 4.8–72.8 *p* < .001). Moreover, we found a strong correlation of MMP-2 expression with fibrinoid necrosis and cellular crescents formation (OR:17.1 95%CI 4.3–67.7 p < .001).

**Conclusions:** MMP expression in renal biopsy of patients with LN is increased and directly related to a highly active inflammatory response. Moreover, stronger MMP expression is associated with higher activity index and a more profound renal dysfunction.

## Introduction

Lupus nephritis (LN) is a serious manifestation of systemic lupus erythematosus (SLE) that contributes significantly to patient morbidity and mortality [1]. Despite the overall improvement in the care of SLE, the prognosis of LN remains unsatisfactory. To improve the outcome of LN further, newer treatment strategies with better efficacy, along with more accurate diagnostic tools is necessary [2]. A newer 2004 approach of LN has led to the International Society of Nephrology (ISN)/Renal Pathology Society (RPS) classification. Apart from the ISN/RPS class of LN, features of reversible (active) or irreversible (chronic) damage on biopsy can predict the prognosis of LN patients. Those with a higher activity index or chronicity index score were more likely to progress to ESRD [3].

Glomerular sclerosis and interstitial fibrosis of the renal parenchyma plays a key role in this unfavorable outcome. While increased synthesis of extracellular matrix (ECM) certainly plays an important role, recent studies have focused on the role of degradative systems. The major physiologic regulators of ECM degradation in the glomerulus are matrix metalloproteinases (MMPs) [4,5].

MMPs are a large family of zinc-dependent matrix-degrading enzymes [5] which include interstitial collagenases (MMP-1, -8, and -13), gelatinases (MMP-2 and -9), stromelysins (MMP-3, -7, and -10) and others. Their proteolytic activity mainly involves the degradation of the ECM components. New evidence indicates that beyond ECM remodeling, MMPs play a key role in inflammation, cellular proliferation, and vascular damage. Therefore, an excess of MMPs may contribute to an inflammatory reaction and result in fibroblast activation, thereby leading to an acceleration of renal fibrosis [5,6].

Several lines of evidence indicate an association between MMPs and many kidney disorders. The type of MMP and the pattern of expression in glomerulus depend on the disease [4].

The activity of MMP is normally inhibited by endogenous specific tissue inhibitors of metalloproteinases (TIMPs) [7], which also have an important role in maintaining mesangial homeostasis [8].

The data on the role of MMPs and TIMPs in the pathogenesis and evolution of renal damage in patients with LN remain very limited. The aim of this study was to examine the expression of MMPs and TIMPs in kidney biopsies of patients with LN and to investigate the relationship between MMPs and renal function at the time of kidney biopsy.

## Patients and methods

### Patients

After reviewing the files of the nephrology department and the archive of the renal pathology department, we obtained a total of 64 cases with a diagnosis of LN. Of the above cases, six were excluded because the biopsy material was inappropriate (lack of glomeruli or generally inadequate biopsy material). Thus, 58 cases (11 males [19%] and 47 females [81%], mean age 34.7 years [SD ± 13.14]) were finally obtained with available formalin-fixed paraffin-embedded tissue suitable for immunohistochemistry, from the original diagnostic renal-biopsy specimen. Four patients (6.9%) had class II LN, 22 patients (37.9%) had class III LN, and 32 patients (55.2%) had class IV LN. 27 patients of them, had also features of membranous LN (class V). The diagnosis of LN was based on tissue studies from light and immunofluorescence microscopy according to the 2004 ISN/RPS classification. Although a revision of ISN/RPS classification has been proposed [9], it is not yet widely applied. However, we have adopted some of the new definitions (e.g., endocapillary hypercellularity instead of endocapillary proliferation). All the biopsies which were performed before 2004 (*n* = 44), were reassessed and reclassified by two different renal-expert pathologists, using the 2004 ISN/RPS classification. Particularly, the individual features of activity and chronicity were reassessed and recorded in detail. Eight histologically normal kidney specimens obtained from eight patients who underwent nephrectomy because of renal carcinoma or renal trauma were used as normal control tissues.

### Antibodies

The immunohistochemical staining was performed on 2.5 μm sections using purified rabbit polyclonal antibodies to human MMP-1, MMP-2, MMP-3, MMP-9, and TIMP-1 which were purchased from Zytomed systems GmbH (Anhaltinerstr. 22, Berlin 14163, Germany). To examine the degree of inflammation, tissue sections were also stained with an antibody against CD68 antigen (DAKO, Carpinteria, CA, USA) as a marker of macrophages.

### Grading of MMPs and TIMP-1 immunostains

All of the glomeruli (3–16) in each section were examined by light microscopy. All the immunostains for MMPs and TIMP-1 were scored without knowledge of the initial histological diagnosis, regarding the ISN/RPS class and the activity and chronicity index or patient’s clinical features. Immunostains were graded on the basis of the intensity of brown staining of the kidney tissue on a scale of 0–3+. A grade of 0–1+ corresponded to absent or weak staining, respectively. A grade of 2+ or 3+ corresponded to increased or significantly increased staining, respectively. For the purposes of comparison, grades 0–1+ were defined as negative and grades 2+–3+ were defined as positive. To assess the variability between MMP grades determined with this technique, grading of the same biopsy samples was repeated on two different days.

### Statistical analysis

Data were expressed as mean ± SD for quantitative variables and as percentages for qualitative variables. The Kolmogorov–Smirnov test was utilized for normality analysis of the quantitative variables. Bivariate analyses were made by using the independent samples t-test or Mann–Whitney test in case of violation of normality and Chi-square test, Fisher’s exact test to analyze the relation between the variables of interest (MMPs) (normal [0–1] *vs.* abnormal [2–3] for MMP1, MMP2, MMP3, TIMP1, and CD68) and the quantitative and qualitative outcome variables (activity and chronicity index and renal function) respectively. Multifactorial logistic and linear regression models were used to identify independent predictors (MMP1, MMP2, MMP3, TIMP1, and CD68) of the qualitative and quantitative outcome variables. All assumptions of linear regression analysis (homoscedasticity, linearity, normality, and independence of error terms, as well as multicollinearity of independent variables) were examined. All tests were two-sided, a *p* value <.05 was used to denote statistical significance. All analyses were carried out using the statistical package SPSS version 17.00 (Statistical Package for the Social Sciences, SPSS Inc., Chicago, IL).

## Results

**Figure 1.**
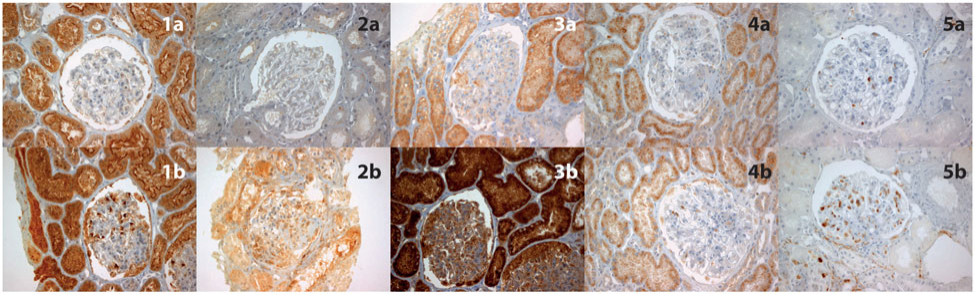
MMPs, TIMP-1 and CD68 expression in LN glomeruli. Whereas MMPs showed a low or negative expression in LN glomeruli with a lower activity index, they were found to be overexpressed in LN glomeruli with higher activity index. In particular: (1a) Low (1+) MMP-1 expression in a class III LN glomerulus with activity index 3, (1b) Stronger (3+) MMP-1 expression in a class IV LN glomerulus with activity index 7, (2a) Negative (0+) MMP-2 staining in a LN glomerulus with activity index 2, (2b) Strong (3+) MMP-2 expression in a class IV LN glomerulus with a crescent and activity index 11, (3a) (1+) MMP-3 expression in a LN glomerulus with activity index 4, (3b) Stronger (3+) MMP-3 expression in a class IV LN glomerulus with activity index 7, (4a) Negative (0+) TIMP-1 staining in a LN glomerulus, 4b) Low (1+) TIMP-1 expression in a LN glomerulus. No significant correlation with any of the activity or chronicity features was detected. (5a) Lack of CD68+ macrophages in glomeruli with lower activity index, (5b) The presence of CD68 + macrophages were observed in cases with a higher activity index score.

On staining for MMP-9, we found no difference between LN and normal glomeruli. We observed a distinct staining pattern for MMP-9, which was consistently expressed in the glomerular basement membrane (GBM), in both normal and LN kidneys. Regarding the TIMP-1, generally we found no significant expression in both normal and LN glomeruli. In some LN glomeruli with stronger expression of TIMP-1, we were unable to show any correlation with activity or chronicity features (Figure 1(4a,4b)).

However, our analysis showed marked differences in MMP-1, MMP-2, and MMP-3 expression between LN and normal glomeruli. Staining for MMP-1, MMP-2, and MMP-3 was negative in all normal glomeruli, while MMP-1, MMP-2, and MMP-3 were strongly expressed in LN glomeruli. More specifically:

### MMP-1

We found that MMP-1 was strongly expressed in LN glomeruli. Moreover, we found a significant correlation with all activity features ([endocapillary hypercellularity OR:7.7 95%CI 2.3–26.3, *p* =.001], [leukocyte influx OR:16.5 95%CI 4.3–62.5 *p* < .001], [subendothelial hyaline deposits, OR:9.8 95%CI 1.1–8.6 *p*=.026], [fibrinoid necrosis and karyorrhexis, OR:17 95%CI 3.4–85 *p* < .001], [cellular crescents, OR:9.5 95%CI 2.3–38.5 *p*=.001], [interstitial inflammation, OR:6 95%CI 1.9–18.8 *p*=.002]). Similarly, strong MMP-1 expression in LN glomeruli was correlated with higher activity index compared with the glomeruli with absent or weak staining (activity index 10.23 ± 2.6 *vs.* 3.96 ± 2.17, *p* < .001) (Table 1 and Figure 1(1a,1b)).

**Table 1.** MMP-1 expression is significantly correlated with all activity features in LN glomeruli.

		MMP-1 (0–1+)		MMP-1 (2–3+)		ΟR (95%CI)	*p* Value
		*ν*	%	*ν*	%		
Endocapillary hypercellularity	0–1+	17	60.7	5	16.7	7.7 (2.3–26.3)	.001
	2–3+	11	39.3	25	83.3		
Leukocyte influx	0–1+	24	85.7	8	26.7	16.5(4.3–62.5)	<.001
	2–3+	4	14.3	22	73.3		
Subendothelial hyaline deposits	0–1+	27	96.4	22	73.3	9.8(1.1–8.6)	.026
	2–3+	1	3.6	8	26.7		
Fibrinoid necrosis and karyorrhexis	0	26	92.9	13	43.3	17.0(3.4–85.0)	<.001
	1–2–3+	2	7.1	17	56.7		
Cellular crescents	0	25	89.3	14	46.7	9.5(2.3–38.5)	.001
	1–2–3+	3	10.7	16	53.3		
Interstitial inflammation	0–1+	21	75.0	10	33.3	6.0(1.9–18.8)	.002
	2–3+	7	25.0	20	66.7		
Glomerular sclerosis	0–1+	18	64.3	21	70.0	0.8(0.3–2.3)	.781
	2–3+	10	35.7	9	30.0		
Fibrous crescents	0–1+	25	89.3	23	76.7	2.5(0.6–11.0)	.301
	2–3+	3	10.7	7	23.3		
Tubular atrophy	0–1+	20	71.4	25	83.3	0.5(0.1–1.8)	.352
	2–3+	8	28.6	5	16.7		
Interstitial fibrosis	0–1+	9	32.1	15	50.0	0.5(0.2–1.4)	.192
	2–3+	19	67.9	15	50.0		

Stronger staining (2+ and 3+) is correlated with higher (2–3+) endocapillary hypercellularity, more intense leukocyte infiltration (2–3+), more subendothelial hyaline deposits (2–3+), fibrinoid necrosis, and karyorrhexis, the presence of cellular crescents (1+, 2+, and 3+) and severe interstitial inflammation (2–3+). Weak staining (0–1+) is associated with lower activity indices (0–1+). On the contrary, strong MMP-1 staining was not correlated with chronicity indices.

N: number of patients; or: odds ratio

### MMP-2

MMP-2, was also strongly expressed in LN glomeruli, but it was significantly correlated only with leukocyte influx (OR:4.9 95%CI 1.6–15.3 *p* < .007), fibrinoid necrosis, and karyorrhexis (OR:17.1 95%CI 4.3–67.7 *p* < .001), cellular crescents (OR:17.1 95%CI 4.3–67.7 *p* < .001) and interstitial inflammation (OR:4.3 95%CI 1.4–13.3 *p*=.015). Regarding the activity index score, stronger MMP-2 expression was correlated with higher activity index compared with the glomeruli with absent or weak staining (activity index 10.55 ± 2.84 *vs.*5.17 ± 3.06, *p* < .001) (Table 2 and Figure 1(2a,2b)).

**Table 2.** Stronger MMP-2 staining (2+ and 3+) is correlated with more intense leukocyte infiltration (2–3+), severe interstitial inflammation (2–3+), and particularly with fibrinoid necrosis and the presence of cellular crescents (1+, 2+ and 3+).

		MMP-2 (0–1+)		MMP-2 (2–3+)		ΟR (95%CI)	*p* Value
		*ν*	%	*ν*	%		
Endocapillary hypercellularity	0–1+	16	44.4	6	27.3	2.1 (0.7–6.7)	.267
	2–3+	20	55.6	16	72.7		
Leukocyte influx	0–1+	25	69.4	7	31.8	4.9 (1.6–15.3)	.007
	2–3+	11	30.6	15	68.2		
Subendothelial hyaline deposits	0–1+	32	88.9	17	77.3	2.4 (0.6–10.0)	.278
	2–3+	4	11.1	5	22.7		
Fibrinoid necrosis and karyorrhexis	0	32	88.9	7	31.8	17.1 (4.3–67.7)	<.001
	1–2–3+	4	11.1	15	68.2		
Cellular crescents	0	32	88.9	7	31.8	17.1 (4.3–67.7)	<.001
	1–2–3+	4	11.1	15	68.2		
Interstitial inflammation	0–1+	24	66.7	7	31.8	4.3 (1.4–13.3)	.015
	2–3+	12	33.3	15	68.2		
Glomerular sclerosis	0–1+	21	58.3	18	81.8	0.3 (0.9–1.1)	.087
	2–3+	15	41.7	4	18.2		
Fibrous crescents	0–1+	32	88.9	16	72.7	3.0 (0.7–12.2)	.156
	2–3+	4	11.1	6	27.3		
Tubular atrophy	0–1+	26	72.2	19	86.4	0.4 (0.1–1.7)	.332
	2–3+	10	27.8	3	13.6		
Interstitial fibrosis	0–1+	14	38.9	10	45.5	0.8 (0.3–2.2)	.784
	2–3+	22	61.1	12	54.5		

As in MMP-1, weak MMP-2 staining (0–1+) is associated with lower activity indices (0–1+). MMP-2 staining was not correlated with chronicity indices.

N: number of patients; or: odds ratio.

### MMP-3

MMP-3 was also strongly expressed in LN glomeruli. In addition, we found a significant correlation with all activity features except for subendothelial hyaline deposits ([endocapillary hypercellularity, OR:18.6 95%CI 4.8–72.8 *p* < .001], [leukocyte influx OR:16.9 95%CI 4.1–69.5 *p* < .001], [subendothelial hyaline deposits, OR:3.1 95%CI 0.6–16.4 *p*=.275], [fibrinoid necrosis and karyorrhexis, OR:6.9 95%CI 1.7–27.6 *p*=.005], [cellular crescents, OR:12.2 95%CI 2.5–60.4 *p*=.001], [interstitial inflammation, OR:4 95%CI 1.3–12.1 *p*=.018]). Similarly, strong MMP-3 expression in LN glomeruli was correlated with higher activity index compared with the glomeruli with absent or weak staining (activity index 9.67 ± 2.78 *vs.* 3.96 ± 2.75, *p* < .001) (Table 3 and Figure 1(3a,3b)).

**Table 3.** Strong MMP-3 staining (2+ and 3+) is correlated with higher (2–3+) endocapillary hypercellularity, more intense leukocyte infiltration (2–3+), with fibrinoid necrosis and the presence of cellular crescents (1+, 2+ and 3+) and severe interstitial inflammation (2–3+).

		MMP-3 (0–1+)		MMP-3(2–3+)		ΟR (95%CI)	*p* Value
		*ν*	%	*ν*	%		
Endocapillary hypercellularity	0–1+	18	72.0	4	12.1	18.6 (4.8–72.8)	<.001
	2–3+	7	28.0	29	87.9		
Leukocyte influx	0–1+	22	88.0	10	30.3	16.9 (4.1–69.5)	<.001
	2–3+	3	12.0	23	69.7		
Subendothelial hyaline deposits	0–1+	23	92.0	26	78.8	3.1 (0.6–16.4)	.275
	2–3+	2	8.0	7	21.2		
Fibrinoid necrosis and karyorrhexis	0	22	88.0	17	51.5	6.9 (1.7–27.6)	.005
	1–2–3+	3	12.0	16	48.5		
Cellular crescents	0	23	92.0	16	48.5	12.2 (2.5–60.41)	.001
	1–2–3+	2	8.0	17	51.5		
Interstitial inflammation	0–1+	18	72.0	13	39.4%	4.0 (1.3–12.1)	.018
	2–3+	7	28.0	20	60.6		
Glomerular sclerosis	0–1+	16	64.0	23	69.7	0.8 (0.3–2.3)	.779
	2–3+	9	36.0	10	30.3		
Fibrous	0–1+	23	92.0	25	75.8	3.7 (0.7–19.5)	.163
Crescents	2–3+	2	8.0	8	24.2		
Tubular atrophy	0–1+	19	76.0	26	78.8	0.9 (0.3–3.0)	.000
	2–3+	6	24.0	7	21.2		
Interstitial fibrosis	0–1+	10	40.0	14	42.4	0.9 (0.3–2.6)	.000
	2–3+	15	60.0	19	57.6		

Weak MMP-3 staining (0–1+) is associated with lower activity indices (0–1+). MMP-3 staining was not correlated with chronicity indices.

N: number of patients; or: odds ratio.

Conversely, we found no significant correlation between any of MMP-1, MMP-2, or MMP-3 expression with any of the glomerular lesions that define chronicity (glomerular sclerosis, fibrous crescents, tubular atrophy, and interstitial fibrosis).

### CD68

Further immunohistochemical analysis showed an increased number of CD68+ cells in cases with higher activity index score (*p* < .001) ((Figure 1(5a,5b)). More specifically, the presence of CD68+ cells was strongly correlated with endocapillary hypercellularity (OR:39.3 95%CI 8.4–183.4 *p* < .001) and leukocyte influx (OR:74.8 95%CI 8.9–643.8 *p* < .001). It showed a weaker (but also significant) correlation with fibrinoid necrosis and karyorrhexis (OR:3.9 95%CI 1.1–14 *p*=.046), cellular crescents (OR:3.9 95%CI 1.1–14 *p*=.046) and interstitial inflammation (OR:3.5 95%CI 1.1–10.6 *p*=.034) (Table 4).

**Table 4.** Strong PGM-1 staining (2–3+) is significantly correlated with higher (2–3+) activity indices.

		PGM-1 (0–1+)		PGM-1 (2–3+)		ΟR (95%CI)	*p* Value
		*ν*	%	*ν*	%		
Endocapillary hypercellularity	0–1+	19	79.2	3	8.8	39.3 (8.4–183.4)	<.001
	2–3+	5	20.8	31	91.2		
Leukocyte influx	0–1+	24	100.0	8	23.5	74.8 (8.9–643.8)	<.001
	2–3+	0	0.0	26	76.5		
Subendothelial hyaline deposits	0–1+	23	95.8	26	76.5	7.1 (0.8–60.9)	.067
	2–3+	1	4.2	8	23.5		
Fibrinoid necrosis and karyorrhexis	0	20	83.3	19	55.9	3.9 (1.1–14.0)	.046
	1–2–3+	4	16.7	15	44.1		
Cellular crescents	0	20	83.3	19	55.9	3.9 (1.1–14.0)	.046
	1–2–3+	4	16.7	15	44.1		
Interstitial inflammation	0–1+	17	70.8	14	41.2	3.5 (1.1–10.6)	.034
	2–3+	7	29.2	20	58.8		
Glomerular sclerosis	0–1+	13	54.2	26	76.5	0.4 (0.1–1.1)	.104
	2–3+	11	45.8	8	23.5		
Fibrous crescents	0–1+	21	87.5	27	79.4	1.8 (0.4–7.9)	.500
	2–3+	3	12.5	7	20.6		
Tubular atrophy	0–1+	16	66.7	29	85.3	0.4 (0.1–1.2)	.118
	2–3+	8	33.3	5	14.7		
Interstitial fibrosis	0–1+	8	33.3	16	47.1	0.6 (0.2–1.7)	.418

It represents the presence of CD68+ cells and is particularly associated with higher endocapillary hypercellularity and more intense leukocyte infiltration. PGM-1 staining was not correlated with chronicity indices.

N: number of patients, or: odds ratio.

### Correlation of MMP expression with renal function and other clinical parameters

Unfortunately, because of the inadequacy of laboratory data and clinical information in the older patients’ records, we were able to obtain laboratory data only from 36 patients (16 patients had eGFR > 60 mL/min, 16 patients had eGFR 30–59 mL/min, and four patients had eGFR <29 mL/min), which showed that the strong expression of MMP-1, MMP-2, and MMP-3 in the glomeruli of patients LN correlated with worse renal function (eGFR) at the time of kidney biopsy. More specifically, the mean eGFR in patients with MMP-1-positive biopsies was 41.17 ± 2.67 mL/min *vs.* 65.54 ± 2.53 in MMP-1-negative biopsies (*p* < .001). Similarly, the mean eGFR in patients with MMP-2-positive biopsies was 40.45 ± 3.33 mL/min *vs.* 63.78 ± 2.62 in MMP-2-negative biopsies (*p* < .001) and finally, the mean eGFR in patients with MMP-3-positive biopsies was 43.23 ± 3.11 mL/min *vs.* 64.51 ± 2.84 in MMP-3-negative biopsies (*p* < .001) (Table 5).

**Table 5.** Clinical and parameters of patients (*N* = 36) in comparison with MMP-1, MMP-2, and MMP-3 expression, at the time of renal biopsy.

*N* = 36	MMP-1 negative	MMP-1 positive	*p*	MMP-2 negative	MMP-2 positive	*p*	MMP-3 negative	MMP-3 positive	*p*
	Mean ± SD	Mean ± SD		Mean ± SD	Mean ± SD		Mean ± SD	Mean ± SD	
Age, years	36.7 ± 13.6	34.8 ± 14	NS	37.4 ± 12.9	34.2 ± 14.5	NS	38.8 ± 13.8	34.7 ± 13.2	NS
eGFR (mL/min)	65.54 ± 2.53	41.17 ± 2.67	<.001	63.78 ± 2.62	40.45 ± 3.33	<.001	64.51 ± 2.84	43.23 ± 3.11	<.001
Anti-ds-DNA (IU/mL)	51.9 ± 29.7	56.9 ± 47.9	NS	49.2 ± 27	60.7 ± 54	NS	58.8 ± 54.9	51.7 ± 30.7	NS
C4 (mg/dL)	8.5 ± 2.5	8.5 ± 3.4	NS	8.2 ± 2.3	8.6 ± 3.7	NS	8.8 ± 3.7	8.2 ± 2.6	NS
Urine protein (g/24 h)	3.2 ± 2.2	3.5 ± 3.2	NS	3.4 ± 2.9	2.7 ± 2.3	NS	2.8 ± 2.2	3.8 ± 3.2	NS
Immunosuppressive therapy (cyclophosphamide or mycophenolate)	None	None	NS	None	None	NS	None	None	NS

Strong expression of MMP-1, MMP-2, and MMP-3 was correlated with worse renal function (eGFR) at the time of kidney biopsy. All the patients of the study where white Caucasians (Greeks) and all the biopsies came from patients who were identified with LN for first time. None of the patients was given immunosuppressive therapy with cyclophosphamide or mycophenolate acid prior to biopsy. Only nine patients were given corticosteroids and/or hydroxychloroquine before the biopsy, because of the extrarenal manifestations of SLE, but it was not correlated with the intensity of the MMP expression.

### Multifactorial logistic regression (forward wald selection)

Multifactorial logistic regression analysis using MMP-1, MMP-2, MMP-3, and PGM-1 as predictive markers, showed a strong correlation of strong MMP-1 expression with subendothelial hyaline deposits (OR:9.8, 95%CI 1.1–84.6 *p*=.038), with fibrinoid necrosis (OR:23.6, 95%CI 2.1–260.5 *p*=.010), and interstitial inflammation (OR:6, 95%CI 1.9–18.8 *p*=.002). Stronger MMP-2 expression was correlated with higher incidence of fibrinoid necrosis (OR:48.7, 95%CI 3.3–720.9 *p*=.005) and cellular crescents (OR:11.8, 95%CI 2.7–50.6 *p*=.001). Stronger MMP-3 expression was correlated with higher incidence of cellular crescents (OR:7.3, 95%CI 1.2–42.1 *p*=.027). Finally, a strong expression of CD68 was correlated with endocapillary hypercellularity (OR:39.3, 95%CI 8.4–183.4 *p* < 001) and leukocyte influx (OR:74.7, 95%CI 8.7–643.8 *p* < .001).

## Discussion

Although important progress has been made regarding the pathogenesis of SLE and LN over the past years, several points about the pathogenic processes that underlie the progressive decay in renal function in LN remain obscure. There are some studies supporting an altered glomerular MMP activity in glomerulopathies and particularly in LN [10]. The role of MMPs is quite different in noninflammatory glomerulopathies and glomerulonephritis. In general terms, a downregulation of MMPs has been associated with progression of noninflammatory diseases such as hypertensive glomerulosclerosis and diabetic nephropathy [4]. On the contrary, in inflammatory glomerular diseases, the increased levels of MMP are generally associated with disease activity and influx of inflammatory cells, and both the level and the duration of MMP elevation determine the extent of glomerular damage [11].

We explored this possibility by studying the expression of MMPs (MMP-1, MMP-2, MMP-3, and MMP-9) in the glomeruli of LN kidneys. In addition, given the predictive value of activity and chronicity index regarding the prognosis of LN patients, we studied the correlation of these MMPs with the activity and chronicity features.

Regarding the MMP-9, although there is some evidence supporting that MMP-9 may be involved in the pathogenesis of human glomerulonephritis [10], we did not find any difference in MMP-9 expression between normal and LN glomeruli. MMP-9 was consistently expressed in the glomerular basement membrane in both normal and LN kidneys. This result might suggest that MMP-9 plays a physiological role in basement membrane turnover.

However, a significant expression of MMP-1, MMP-2, and MMP-3 in LN glomeruli emerged in our study. Moreover, we found a strong correlation of MMP-1, MMP-2, and MMP-3 expression with the various activity features of LN and a higher activity index score.

Particularly, MMP-1 showed a notable correlation with the leukocyte influx as it is shown by the high OR (16.5). Among the patients with weak MMP-1 expression (*n* = 28), 24 patients (85.7%) showed a low-grade leukocyte influx, while only four patients (14.3%) had a significant leukocyte influx. On the contrary, among the patients with strong MMP-1 expression (*n* = 30), 22 patients (73.3%) had a significant leukocyte influx, while only eight patients (26.7%) had low-grade leukocyte influx (OR:16.5 95%CI 4.3–62.5 and *p* < .001).

Regarding MMP-3, the results are quite similar. In particular, we have noticed a strong correlation with glomerular hypercellularity (*p* < .001).

These findings suggest that high MMP-1 and MMP-3 expression in LN glomeruli is more compatible with an exudative form of glomerulonephritis in which leukocyte accumulation lead to endothelial cell injury and capillary wall destruction. MMP-1 and MMP-3 may be secreted by activated infiltrating cells (monocytes/macrophages and neutrophils) [12] through activation of the Toll-like receptor 9 signaling pathway [13,14]. This is in accordance with our finding of increased number of CD68+ cells within the glomeruli with higher activity index score (hypercellularity and leukocyte influx) and strong MMP expression.

It is known that MMP-1 is involved in the breakdown of the main collagens (types I–III) of the interstitial connective tissue as well as laminin and tenascin [15–17] and MMP-3 have a broad substrate specificity including collagen IV, proteoglycans, fibronectin, and laminin [17,18]. Since collagen IV, laminin, and fibronectin are the main constituents of mesangial matrix and glomerular basement membrane, it appears that MMP-1 and MMP-3 are capable of mediating changes in glomerular basement membrane by cleavage of particular collagen fragments and lead to the exposure of highly immunoreactive epitopes, increasing this way the production of autoantibodies against matrix structures [19,20].

The gelatinase MMP-2 is also involved in the catabolism of collagen IV and laminin, the most important components of mesangial matrix and glomerular basement membrane. Strong MMP-2 expression showed a particular correlation with fibrinoid necrosis (*p* < .001) and the presence of cellular crescents (OR:17.1 95%CI 4.3–67.7 and *p* < .001). Among the patients with strong MMP-2 staining (*n* = 22), 15 patients (68.2%) had fibrinoid necrosis and cellular crescents, while only seven patients (31.8%) had no fibrinoid necrosis or cellular crescents.

It is known that besides ECM degradation, MMPs play a key role in inflammation and cellular proliferation and may also influence ECM turnover via the regulation of certain growth factors. Thus, our finding may suggest that MMP-2 could play an important role in crescents formation. Moreover, MMP-2 expression could contribute to prediction of renal outcome in crescenting LN, as the presence of cellular crescents and fibrinoid necrosis are associated with a worse renal prognosis. Triantafyllopoulou et al. found that macrophages directly contribute to the elevated MMP-2 expression in LN [21].

Multifactorial logistic regression revealed a potential predictive value of MMP-1, MMP-2, and MMP-3 regarding subendothelial hyaline deposits, fibrinoid necrosis, cellular crescents, and interstitial inflammation.

Increased expression of the MMPs is often found to be accompanied by a compensatory increase in the levels of one or more of the TIMPs. The net result of these opposing stimuli could determine the final effect on the ECM turnover. There is also additional data in experimental nephritis showing that MMP inhibition attenuates glomerular lesions [22], suggesting a role for the MMP inhibitors in the treatment of acute glomerulonephritis [22–24]. Regarding TIMP-1, although there is evidence supporting increased plasma and urinary concentrations in patients with renal disease, the data are inadequate. In our study, although we found a stronger expression of TIMP-1 in some LN glomeruli, we were unable to determine any significant correlation with the various features of disease activity. This could be due to the fact that TIMP-1 is the main inhibitor of MMP-9, which was equally expressed in normal and LN glomeruli in our study.

In addition to correlating the expression of MMPs with the activity index of LN, the association of stronger MMP-1, MMP-2, and MMP-3 expression with worse renal function at the time of kidney biopsy, indicates that these proteolytic enzymes play a pivotal role in mesangial matrix and glomerular basement membrane damage, thus, contributing to the impairment of renal function.

Our findings are in accordance with previous studies showing increased levels of MMP-1, MMP-2, and MMP-3 on mice with experimental LN. Data from human studies also showed that elevated MMP levels are associated with a highly active inflammatory response associated with glomerular damage [10]. To our knowledge, our study is the first study in LN including such a large number of biopsy samples, and also the first study in LN correlating the MMP expression with the various activity features and renal function.

There is evidence that the MMP activity may be different even in the different stages of the same renal disease. MMPs may contribute to GBM degradation in the early stages, while they play a pivotal role in the removal of ECM associated with scarring and fibrosis at later stages [25]. In our study, we found no particular correlation between the expression of MMP-1, MMP-2, MMP-3, and MMP-9 and the various classes of LN. In addition, the lack of any significant correlation between MMP activity and the chronicity features, could mean that although MMPs play a certain role in cell proliferation and cell infiltration, their role in the EC degradation and removal in the advanced stages of LN is limited.

To successfully manage LN, it is important to be able to accurately distinguish the onset of renal disease, to determine the disease activity and finally to distinguish active nephritis from chronic kidney damage. The identification of LN biomarkers could help in accurate assessment of disease activity and severity and enable the physician to individualize the therapy [26]. Although some studies reported some biomarkers that correlate with the histological findings in LN [27], no reliable biomarker which could represent a specific histological lesion and with a clinical value has been emerged yet [26]. In this manner, our study could provide additional data regarding the MMPs expression in LN and their significance as a potential histologic biomarker with diagnostic and prognostic value.

In conclusion, MMP expression in renal biopsy of patients with LN is increased and directly related to a highly active inflammatory response. Moreover, stronger MMP expression is associated with higher activity index and a more profound renal dysfunction at the time of renal biopsy. Particularly, strong MMP-2 expression appears to be highly correlated with the presence of cellular crescents and fibrinoid necrosis. Our findings may contribute to the interpretation of the pathogenesis of renal damage in LN and could provide a potential marker for the activity of the disease.

### Limitations of the study

This is a cross-sectional study regarding the expression of MMPs in LN and their correlation with the activity and chronicity index and renal function at the time of renal biopsy. Many biopsy specimens were retrieved from the archive of the renal pathology department and concerned patients who underwent a renal biopsy many years ago. So unfortunately, information regarding their subsequent course was not sufficient. A prospective study is required to confirm the predictive value of MMPs (besides their correlation with a higher activity index and a worse renal function at the time of kidney biopsy) in the outcome of renal function.

## References

[1] Waldman M, Appel GB Update on the treatment of lupus nephritis. Kidney Int. 2006;70:1403–1412. 16929249 10.1038/sj.ki.5001777

[2] Misra R, Gupta R Biomarkers in lupus nephritis. Int J Rheum Dis. 2015;18:219–232. 25884459 10.1111/1756-185X.12602

[3] Weening JJ, D’Agati VD, Schwartz MM, et al. The classification of glomerulonephritis in systemic lupus erythematosus revisited. Kidney Int. 2004;65:521–530. 14717922 10.1111/j.1523-1755.2004.00443.x

[4] Lenz O, Elliot SJ, Stetler-Stevenson WG Matrix Metalloproteinases in renal development and disease. J Am Soc Nephrol. 2000;11:574–581. 10703682 10.1681/ASN.V113574

[5] Tan RJ, Liu Y Matrix metalloproteinases in kidney homeostasis and diseases. Am J Physiol Renal Physiol. 2012;302:F1351–F1361. 22492945 10.1152/ajprenal.00037.2012 PMC3774496

[6] Liu Y Cellular and molecular mechanisms of renal fibrosis. Nat Rev Nephrol. 2011;7:684–696. 22009250 10.1038/nrneph.2011.149 PMC4520424

[7] Bode W, Fernandez-Catalan C, Tschesche H, et al. Structural properties of matrix metalloproteinases. Cell Mol Life Sci. 1999;55:639–652. 10357232 10.1007/s000180050320 PMC11146962

[8] Keeling J, Herrera GA Matrix metalloproteinases and mesangial remodelling in light chain-related glomerular damage. Kidney Int. 2005;68:1590–1603. 16164636 10.1111/j.1523-1755.2005.00571.x

[9] Bajema IM, Wilhelmus S, Alpers CE, et al. Revision of the international society of nephrology/renal pathology society classification for lupus nephritis: clarification of definitions and modified National institutes of health activity and chronicity indices. Kidney Int. 2018;93:789–796. 29459092 10.1016/j.kint.2017.11.023

[10] Urushihara M, Kagami S, Kuhara T, et al. Glomerular distribution and gelatinolytic activity of matrix metalloproteinases in human glomerulonephritis. Nephrol Dial Transplant. 2002;17:1189–1196. 12105240 10.1093/ndt/17.7.1189

[11] Nakamura T, Ebihara I, Tomino Y, et al. Effect of a specific endothelin A receptor antagonist on murine lupus nephritis. Kidney Int. 1995;47:481–489. 7723234 10.1038/ki.1995.61

[12] Gill SE, Parks WC Metalloproteinases and their inhibitors: regulators of wound healing. Int J Biochem Cell Biol. 2008;40:1334–1347. 18083622 10.1016/j.biocel.2007.10.024 PMC2746915

[13] Lim EJ, Lee SH, Lee JG, et al. Toll-like receptor 9 dependent activation of MAPK and NF-kB is required for the CpG ODN-induced matrix metalloproteinase-9 expression. Exp Mol Med. 2007;39:239–245. 17464186 10.1038/emm.2007.27

[14] Merrell MA, Ilvesaro JM, Lehtonen N, et al. Toll-like receptor 9 agonists promote cellular invasion by increasing matrix metalloproteinase activity. Mol Cancer Res. 2006;4:437–447. 16849519 10.1158/1541-7786.MCR-06-0007

[15] Gagliano N, Arosio B, Santambrogio D, et al. Age-dependent expression of fibrosis-related genes and collagen deposition in rat kidney cortex. J Gerontol A Biol Sci Med Sci. 2000;55:B365–B372. 10952357 10.1093/gerona/55.8.b365

[16] Gross J How tadpoles lose their tails: path to discovery of the first matrix metalloproteinase. Matrix Biol. 2004;23:3–13. 15172033 10.1016/j.matbio.2004.01.003

[17] Nagase H Substrate specificity of MMPs In: Clendeninn NJ, Appelt K, editors. Matrix metalloproteinase inhibitors in cancer therapy. Berlin, Germany: Springer-Science Business media, LLC; 2001 p. 39–66.

[18] Kim EM, Hwang O Role of matrix metalloproteinase-3 in neurodegeneration. J Neurochem. 2011;116:22–32. 21044079 10.1111/j.1471-4159.2010.07082.x

[19] Nelissen I, Martens E, Van den Steen PE, et al. Gelatinase B/matrix metalloproteinase-9 cleaves interferon-beta and is a target for immunotherapy. Brain. 2003;126:1371–1381. 12764058 10.1093/brain/awg129

[20] Opdenakker G, Dillen C, Fiten P, et al. Remnant epitopes, autoimmunity and glycosylation. Biochim Biophys Acta. 2006;1760:610–615. 16439062 10.1016/j.bbagen.2005.12.014

[21] Triantafyllopoulou A, Franzke CW, Seshan SV, et al. Proliferative lesions and metalloproteinase activity in murine lupus nephritis mediated by type I interferons and macrophages. Proc Natl Acad Sci USA. 2010;107:3012–3017. 20133703 10.1073/pnas.0914902107 PMC2840310

[22] Steinmann-Niggli K, Ziswiler R, Kung M, et al. Inhibition of matrix metalloproteinases attenuates anti-Thy1.1 nephritis. J Am Soc Nephrol. 1998;9:397–407. 9513901 10.1681/ASN.V93397

[23] Goss KJ, Brown PD, Matrisian LM Differing effects of endogenous and synthetic inhibitors of metalloproteinases on intestinal tumorigenesis. Int J Cancer. 1998;78:629–635. 9808534 10.1002/(sici)1097-0215(19981123)78:5<629::aid-ijc17>3.0.co;2-8

[24] Lombard MA, Wallace TL, Kubicek MF, et al. Synthetic matrix metalloproteinase inhibitors and tissue inhibitor of metalloproteinase (TIMP)-2, but not TIMP-1, inhibit shedding of tumor necrosis factor-alpha receptors in a human colon adenocarcinoma (Colo 205) cell line. Cancer Res. 1998;58:4001–4007. 9731514

[25] Ronco P, Chatziantoniou C Matrix metalloproteinases and matrix receptors in progression and reversal of kidney disease: therapeutic perspectives. Kidney Int. 2008;74:873–878. 18650796 10.1038/ki.2008.349

[26] Birmingham DJ, Merchant M, Waikar SS, et al. Biomarkers of lupus nephritis histology and flare: deciphering the relevant amidst the noise. Nephrol Dial Transplant. 2017;32:i71–i79. 28391335 10.1093/ndt/gfw300 PMC5837441

[27] Mok CC Biomarkers for lupus nephritis: a critical appraisal. J Biomed Biotechnol. 2010;2010:638413. 20414362 10.1155/2010/638413 PMC2857808

